# ICU mortality prediction via multimodal learning on MIMIC-IV clinical data

**DOI:** 10.1093/bioadv/vbag214

**Published:** 2026-08-05

**Authors:** Alberto Falcone, Agostino Forestiero, Rocco Malivindi, Marzia Settino

**Affiliations:** Institute for High Performance Computing and Networking, Italian National Research Council (CNR-ICAR), Rende 87036, Italy; Institute for High Performance Computing and Networking, Italian National Research Council (CNR-ICAR), Rende 87036, Italy; Department of Pharmacy, Health and Nutritional Sciences, University of Calabria, Rende 87036, Italy; Clinical Laboratory Unit, Azienda Ospedaliera di Cosenza “Annunziata”, Cosenza 87100, Italy; Institute for High Performance Computing and Networking, Italian National Research Council (CNR-ICAR), Rende 87036, Italy

## Abstract

**Purpose:**

This study proposes a multimodal framework for retrospective short-term mortality prediction in intensive care unit patients by integrating structured admission data with semantic information extracted from discharge summaries. The objective is to improve patient characterization by combining complementary clinical data that are often analyzed separately.

**Methods:**

Structured inputs, including demographics, administrative records, laboratory results, and clinical severity scores, were merged with semantic representations of clinical notes produced by a BERT-based encoder. The resulting embeddings were reduced in dimensionality and clustered with K-Means to obtain categorical semantic features reflecting thematic similarity among notes. These semantic cluster identifiers were then concatenated with tabular variables to create a unified multimodal representation, which was used to train five classical machine learning classifiers.

**Results:**

The integration of semantic features consistently improved predictive performance compared with models trained only on structured data. Among the evaluated algorithms, Random Forest achieved the best discrimination, with an area under the receiver operating characteristic curve of 0.940 in the multimodal setting.

**Conclusion:**

Combining structured clinical variables with unstructured admission notes enhances short-term mortality prediction and supports the development of interpretable multimodal decision-support tools for intensive care settings.

## 1 Introduction

Developing prognostic models to assess the risk of adverse outcomes in ICU has long been a key focus in critical care medicine. The ICU is a specialized hospital department where patients receive continuous and intensive monitoring due to critical health conditions. Despite advances in critical care, mortality rates remain reasonably high, especially during the first 30 days post-discharge (Gonçalves-Pereira *et al*. 2023, Halac *et al*. 2025). In the following, short-term mortality denotes mortality occurring during the ICU stay or within 30 days after hospital discharge.

Enhancing short-term mortality prediction remains challenging, as it requires identifying high-risk subjects who may benefit from tailored treatment strategies to improve survival.

Several scoring systems have been developed to predict the prognosis and mortality risk of ICU patients based on their baseline health characteristics and clinical parameters collected within the first 24 hours of ICU admission (Vincent *et al.* 1996b).

The ever-increasing adoption of Electronic Health Records (EHRs) has transformed modern healthcare systems. EHRs have been designed to collect and store a wide range of patient data, including demographic details, diagnoses, laboratory results, prescriptions, radiological images, and clinical notes (https://www.who.int/data/gho/indicator-metadata-registry/imr-details/4791). EHRs have improved the accessibility, integrity, and quality of patient information by enabling automated data entry and standardized formatting. However, human-driven analysis of large-scale clinical data extracted from EHRs remains time-consuming and highly reliant on expert knowledge, which can introduce subjectivity and bias. To address these limitations, there is growing interest in employing ML methods to EHRs for various clinical tasks, such as predicting prognosis, disease inference, and supporting diagnostic decisions (Shickel *et al*. 2018). Although medical expertise and clinical skills cannot be replaced, ML can serve as a valuable support tool by improving the decision-making process and reducing risks in medical practice. Classical machine learning (CML) models, including Support Vector Machines (SVM), Random Forest (RF), Naive Bayes (NB), Generalized Linear Models (GLM), and recursive partitioning and regression trees (RPART), typically use structured data for predictive tasks, overlooking the potential of unstructured information, which accounts for approximately 80% of EHRs. Structured data refers to information that is organized into standardized formats, such as tables or predefined fields, typically including laboratory results, vital signs, medication codes, clinical scores, and diagnostic classifications. In contrast, unstructured data refers to free-text clinical documentation, often providing detailed contextual and patient-specific information that structured data fails to capture. Structured data is readily analyzable and easily integrated into computational pipelines, whereas unstructured data requires advanced techniques to be effectively utilized. As unstructured data, clinical notes offer valuable information that can be effectively analyzed using Natural Language Processing (NLP) (Supriyono *et al.* 2024). Despite their potential, the accessibility of EHR data remains a significant challenge, primarily due to privacy concerns, interoperability issues, institutional access restrictions, safety considerations, and data heterogeneity. In this context, the Medical Information Mart for Intensive Care (MIMIC) (https://physionet.org/content/mimiciv/3.1/) project provides a publicly available, large-scale database of de-identified EHRs data. Building upon this resource, this study proposes a hybrid framework for processing multimodal clinical data from the MIMIC, providing a more comprehensive representation of each patient’s health condition and clinical trajectory.

The proposed framework is termed as *multimodal* because it integrates structured and unstructured data sources, and *hybrid*, as it combines different methodological approaches, namely CML techniques and Large Language Models (LLMs). The goal is to exploit the complementary strengths of multimodal data to improve the reliability of short-term mortality prediction in ICU patients.

This paper is summarized as follows: Section 2 provides background information; Section 3 provides a brief overview of the related works; Section 4 presents the proposed hybrid framework; Section 5 describes the experimental setup; and finally, Section 6 discusses the results and concludes the paper.

## 2 Background

Retrospective medical data analysis has significant potential to develop predictive models that enhance patient care. In this context, the MIMIC database has become a cornerstone resource for advancing ML applications in healthcare, particularly in areas such as patient outcome prediction, disease progression monitoring, and clinical decision-support systems.

The MIMIC database includes comprehensive and de-identified clinical data from ICU patients and a rich collection of clinical notes that provide detailed insights into patients’ conditions, treatments, and clinical progress. Data from MIMIC-IV can be used to calculate several widely used illness severity scores, such as the Logistic Organ Dysfunction System (LODS), Simplified Acute Physiology Score (SAPS), Sepsis-related Organ Failure Assessment (SOFA), and Charlson Comorbidity Index (CCI), which are typically used for risk stratification of ICU patients.

These scoring systems are transparent and interpretable and have been widely validated in clinical practice. However, several studies have shown that these traditional scoring systems often have unsatisfactory accuracy in predicting actual mortality rates, probably due to fixed logic that cannot be adapted to unstructured and complex clinical patterns (Liu *et al*. 2022).

Although multimodal data offer a promising solution to this limitation, their processing remains challenging, primarily due to the frequent use of medical abbreviations in clinical notes, in which healthcare professionals often report drug names, diseases, and other clinical terms (Li *et al*. 2022b, Fan *et al*. 2023).

The variability of these abbreviations across different medical specialties and EHR systems further complicates their interpretation and analysis (Li *et al*. 2022a). In addition, clinical notes often include non-standardized language (e.g. varying terms like “myocardial infarction” vs. “heart attack”), misspellings, irrelevant or redundant content, and fragmented grammar, all of which complicate automated processing. To exploit complementary information from clinical notes, we propose a hybrid multimodal framework that integrates quantitative and semantic data to improve predictive performance. The well-established interest in short-term mortality prediction has driven the development of several ML-based approaches with promising results. The following paragraphs outline the core components of the proposed hybrid framework, which serve as the foundation for understanding the subsequent sections of the paper.

### 2.1 Large language models

LLMs are advanced NLP techniques based on DL architectures designed to understand and generate human-like sentences by identifying complex linguistic patterns within the text. Usually, they are pretrained on large-scale text corpora to generate language that resembles human-produced text. Their explicit objective is to successfully predict the next word or a missing word in a sentence (Clusmann *et al*. 2023). While originally trained for language modeling, LLMs have demonstrated strong potential across a range of tasks, including pattern recognition in images and the analysis of audio and video data. LLMs have proven valuable in streamlining administrative workflows such as billing, appointment scheduling, and claims processing, thereby improving healthcare efficiency and enabling professionals to focus more on patient care. LLMs also have shown great promise in enhancing clinical decision support, by enabling more accurate, data-driven, and context-aware diagnostic and therapeutic recommendations (Busch *et al*. 2025).

LLMs have shown substantial promise in various healthcare domains, such as clinical decision support, diagnostic reasoning, treatment planning, medical documentation, and biomedical research (Zhang *et al*. 2025). Domain-specific LLMs, such as BioBERT and ClinicalBERT, represent promising approaches for clinical text analysis, medical question answering, and named entity recognition (NER) tasks (Tian *et al*. 2023).

Despite their promise, LLMs are typically difficult to interpret (a.k.a. “black-box”), require substantial amounts of data and computational resources and may inherit systematic biases embedded in the training set. LLMs can be broadly categorized into two types: (i) *general-purpose*, which are trained on broad textual data such as news articles and Wikipedia; and (ii) *domain-specific*, which are fine-tuned on specialized corpora (e.g. PubMed) for tasks within a particular domain, like medicine. General-purpose LLMs, like Generative Pre-trained Transformer (GPT), are trained on diverse textual sources, making them highly versatile across a broad range of NLP tasks. In contrast, domain-specific models, while more effective in medical contexts, tend to suffer from limited generalizability and require access to high-quality, specialized datasets, making them more difficult to train, maintain, and deploy across diverse healthcare scenarios.

The *Bidirectional Encoder Representations from Transformers (BERT)* is a general-purpose LLM that uses bidirectional context to enhance performance on NLP tasks, i.e. it captures a word’s meaning by considering context from both directions in a sentence. Unlike traditional models that read text in a single direction, BERT captures the meaning of each word by simultaneously considering the surrounding context from both the left and right sides of the sentence. This characteristic makes it particularly effective for understanding language structures and resolving ambiguities (Zafarmomen and Samadi 2025). The architecture of BERT is structured into four components: (i) *Tokenizer*, which converts the input text into a sequence of sub-word units known as *tokens*. It also introduces the special token [CLS] at the beginning of the sequence that serves as a summary representation of the entire sequence and is commonly used in downstream classification tasks; (ii) *Embedding Layer*, for each input token, it generates an embedding that represents both the specific token and its position within the sequence; (iii) *Encoder (Transformer Layers)*, it involves a stack of transformer layers that apply self-attention mechanisms to produce contextualized embeddings, capturing the semantic relation between tokens across the entire sequence in both directions; and (iv) *Task Specific Head*, it consists of one or more layers designed to generate task-specific predictions from the contextualized embeddings produced by the encoder.

### 2.2 Medical information mart for intensive care

MIMIC-IV (the latest version of the MIMIC database) is a relational database containing retrospective de-identified health-related information on hospital stays of more than 60 000 patients admitted to the Beth Israel Deaconess Medical Center in Boston, MA, USA (Johnson *et al*. 2023). Specifically, it contains data for approximately 24.5% of patients admitted to ICU and 75.5% of patients admitted to the emergency department (Goldberger *et al*. 2000, Johnson *et al*. 2024). The MIMIC-IV database encompasses a wide range of information, including administrative records, demographics, laboratory test results, vital signs (e.g. heart rate, blood pressure, respiration rate, and temperature), and clinical notes.

The MIMIC-IV database is characterized by a relational structure, categorized into distinct “modules” based on the origin and type of the data. The primary modules of MIMIC-IV include: *hosp*, *icu*, and *note*. The *hosp* module contains data sourced from the hospital-wide EHR system, the *icu* module includes data from ICU clinical information system known as “MetaVision,” and the *note* module provides clinical notes and free-text documentation. In the MIMIC-IV database, two key identifiers—*subject_id* and *hadm_id—*are consistently included across all tables. *Subject_id* is a unique identifier for each patient, enabling linkage to related demographic data, whereas *hadm_id* corresponds to a specific hospital admission, enabling tracking of patient-specific admissions.

Access to the MIMIC-IV database requires users to confirm their identity, complete a training course, and pass a qualification test. Upon completion, users must register, obtain approval from the database administrator staff to extract data for research purposes, and sign a data use agreement. The MIMIC code repository facilitates public discussion and code sharing, promoting transparency, reproducibility, and collaboration between research groups (Johnson *et al.* 2018).

### 2.3 Clinical scores

Illness severity and comorbidity scores are numerical measures used to assess the severity of the disease of a patient and the overall burden of comorbidities (Vincent *et al*. 2000). Typically, illness severity and comorbidity scores are combined to comprehensively assess the patient’s prognosis. In this study, we use the term *clinical scores* to denote both illness severity and comorbidity scores. In particular, illness severity scores are derived from structured variables such as laboratory measurements, vital signs, and indicators of organ function, and they are calculated using predefined formulas. Table 1 presents three of the four clinical scores used in this study, along with the primary variables involved in their calculation. Primary variables refer to the raw and directly measured or observed values collected from a patient (e.g. vital signs, laboratory results, and demographic data). Unlike the other clinical scores, the fourth score, CCI, used in this study is derived from diagnosed comorbidities, such as Myocardial infarction, Chronic pulmonary disease, Diabetes, Tumor, Leukemia, and AIDS, rather than from physiological measurements. Details of each score are provided below.

**Table 1:** Overview of the primary variables utilized to compute the illness severity scores used in this study.

Severity score	Embedded variables
**SOFA**	gcs, map, FiO_2_, ventilation status, creatinine, bilirubin, PaO_2_, platelets, dopamine, dobutamine, epinephrine, norepinephrine, urine output
**LODS**	GCS, heart rate, systolic blood pressure, ventilation/CPAP status, urine output, BUN, WBC, bilirubin, creatinine, PT, platelets, PaO_2_ with associated FiO_2_
**SAPS II**	age, GCS, heart rate, systolic blood pressure, temperature, ventilation/CPAP status, urine output, PaO_2_/FiO_2_ ratio, BUN, WBC, potassium, sodium, HCO_3_

Acronyms: GCS, Glasgow Coma Scale; MAP, Mean Arterial Pressure; FiO_2_, Fraction of Inspired Oxygen; PaO_2_, Partial pressure of arterial oxygen; CPAP, Continuous Positive Airway Pressure; BUN, Blood Urea Nitrogen; WBC, White Blood Cell count; PT, Prothrombin Time; HCO_3_, Bicarbonate.

#### 2.3.1 Sequential organ failure assessment (SOFA)

It describes quantitatively and objectively the degree of organ failure over time in groups of patients or even individual patients (Vincent *et al*. 1996). It focuses on six organ systems: respiratory, coagulation, hepatic, cardiovascular, central nervous, and renal. Each of six organ systems is independently assigned a score of 1 to 4. The total SOFA score, obtained by summing individual organ scores, increases with the severity of the patient’s condition. The score is typically calculated within the first 24 hours of a patient’s ICU stay and is defined as:


(1) $$\text{SOFA}\_{\text{Score}}_{T}=\sum_{i=1}^{6}\;\text{SOFA}\_{\text{Score}}_{i}$$

where, each $\mathrm{SOFA}\_\mathrm{Scor}e_{i}$ corresponds to the value assigned to one of the six organ systems.

#### 2.3.2 Logistic organ dysfunction system (LODS)

This score provides an objective tool for assessing the severity of organ dysfunction. It is calculated using a total of 12 variables—two for each of six organ systems. Scores (ranging from 0 to 5) are assigned during the first 24 hours of ICU admission to each organ system based on the severity of dysfunction (Le Gall *et al*. 1993, Gall *et al*. 1996). LODS is the sum of these six subscores and is defined as:


(2) $$\text{LODS}=\sum_{i\in \{n,c,r,p,h,l\}}{\text{Score}}_{i}$$

where ${\text{Score}}_{i}$ denotes the score for organ system *i*, with i=n (neurologic), *c* (cardiovascular), *r* (renal), *p* (pulmonary), *h* (hematologic), and *l* (hepatic/liver).

#### 2.3.3 Simplified acute physiology score II

It is a weighted sum of 17 clinical variables measured during the first 24 hours after admission to the ICU. The resulting score is then converted into a probability of ICU mortality using the following logistic function:


(3) $$\text{logit}(p)=\alpha_{0}+\alpha_{1}{\text{SAPS}}_{\text{II}}+\alpha_{2}\;\text{log}({\text{SAPS}}_{\text{II}}+1)$$

where $\alpha^{0},\alpha^{1},\alpha^{2}$ are the model parameters, and SAPS_II_ is the score obtained by summing the weighted contributions of the 17 clinical variables including physiological measurements, laboratory values, age and type of admission (elective or emergency) (Le Gall *et al*. 2005).

#### 2.3.4 Charlson comorbidity index (CCI)

It is a validated scoring system used to estimate the burden of comorbidity and its impact on patient outcomes. These comorbidities are typically identified using ICD-9 or ICD-10 diagnosis codes extracted from EHRs (Quan *et al*. 2005). These codes are defined by the International Classification of Diseases (ICD) developed by the World Health Organization (WHO) for coding and classifying the diseases, symptoms, injuries, and other health-related conditions (Charlson *et al*. 1987, 1994, Quan *et al*. 2005). The comprehensive CCI score is calculated by summing the weights of all identified comorbidities and is defined as:


(4) $$\text{CCI}=\sum_{j=1}^{N}w_{j}\cdot C_{j}$$

where *N* is the total number of comorbidity conditions considered, $w_{j}\in \{1,2,3,6\}$ is the weight assigned to condition *j*, and $C_{j}\in \{0,1\}$ indicates whether the condition is absent or present, respectively.

## 3 Related work

Several studies have proposed ML-based approaches to predict clinical outcomes, with some utilizing data from the MIMIC. In (Hou *et al*. 2020, Yang *et al*. 2023), the authors used the MIMIC-III database to develop models for predicting short- and intermediate-term mortality in ICU patients. Both studies demonstrate that the XGBoost model achieves high predictive performance, effectively assisting clinicians in the early identification of high-risk patients. Similarly, an ML-based approach was also adopted in (Hempel *et al*. 2023), where the MIMIC-IV database was used to train models for predicting the length of stay (LOS) of ICU patients, with the aim of supporting resource planning, optimizing bed allocation, and improving patient flow. In recent years, there has been a growing interest in using LLMs to extract insights from unstructured data embedded in EHRs. In this context, several domain-specific LLMs, referred to as clinical LLMs, have been developed to support tasks in the healthcare field. Notable examples include BioBERT, the first BERT-based model pre-trained on large-scale biomedical text corpora (Lee *et al*. 2020), and ClinicalBERT, which was pre-trained on clinical notes from the MIMIC-III database (Kollapally and Geller 2023). These models have shown strong performance across various biomedical NLP tasks, such as diagnosis classification, document categorization, and the detection of meaningful relationships between clinical entities (e.g. drug-disease associations).

Other notable models include Asclepius-R, which was trained on MIMIC-III discharge summaries to generate accurate and context-aware responses to clinical prompts, and LLaMACare, designed to enhance accuracy and relevance in medical NLP tasks (Kweon *et al*. 2024, Li *et al*. 2024). Furthermore, disease-specific LLMs, a subcategory of clinical LLMs, have been introduced to handle specialized clinical tasks with greater precision. One such example is VTE-BERT (Lam *et al*. 2024), a clinical LLM designed to identify cases of venous thromboembolism (VTE) through the labeling of radiology reports from computed tomography pulmonary angiography (CTPA). VTE-BERT was fine-tuned using data extracted from the MIMIC-IV database. Concurrent with the advancement of clinical LLMs, the research community has placed growing emphasis on reproducibility and standardization in medical NLP. In (Lovon-Melgarejo *et al*. 2024), the authors introduced a publicly available Hugging Face datasets (https://huggingface.co/docs/datasets/index), which enables reproducible use of the MIMIC-IV benchmark for health-related tasks. This initiative underscores the crucial role of accessible, well-structured datasets in driving progress in medical research (Omiye *et al*. 2024). Although the application of ML and LLMs in medicine is gaining increasing attention, the potential to leverage multimodal data in healthcare analytics remains relatively underexplored. For instance, in the works (Wu *et al*. 2024, Zhang *et al*. 2024), the authors showed that combining structured and unstructured data improves predictive accuracy; however, these findings are limited to specific scenarios, mortality prediction of patients with heart failure and severity assessment in emergency department admissions, respectively. Similarly, in the work (Chiu *et al*. 2024), the authors proposed a hybrid model that combines BERTopic—a clinical LLM- with Long Short-Term Memory (LSTM) networks to predict ICU readmission. However, this work does not integrate clinical scores such as SOFA, SAPS II, or LODS, which are key indicators of disease severity and organ dysfunction. The work Soenksen *et al*. (2022) introduces HAIM (Holistic AI in Medicine), a multimodal framework built on MIMIC-IV. However, the proposed framework relies mainly on complex deep learning architectures, which limit interpretability and clinical transparency. The systematic review Huang *et al*. (2020) provides a comprehensive taxonomy of multimodal fusion strategies, including early, late, and hybrid fusion approaches, thereby offering a useful framework to contextualize and motivate the architectural design adopted in the proposed Feature Fusion module.

Overlooking clinically validated severity scores implies the loss of a well-established clinical reference for overall patient severity. Moreover, while 30-day readmission is an important metric for healthcare system efficiency, it is influenced by multiple external factors (e.g. discharge processes, comorbidities, and post-discharge follow-up) and may not directly reflect the the underlying short-term clinical deterioration of ICU patients. Additionally, most existing studies focus on visit-level in-hospital mortality prediction using end-to-end DL architectures (Grnarova *et al.* 2016, Lyu *et al*. 2023, Gana *et al*. 2025), often providing limited interpretability of the learned clinical representations.

To address this gap, we introduce a hybrid framework for admission-level short-term mortality modeling in ICU patients that integrates structured clinical variables, including clinically validated severity scores (e.g. SOFA and SAPS II), together with unstructured discharge clinical notes. Differently from previous works, our framework does not rely on end-to-end DL prediction architectures. Instead, although LLMs are based on DL, their role is limited to generating semantic representations of clinical notes, which are subsequently transformed into explicit categorical semantic features through clustering. These interpretable semantic features are then integrated with structured clinical data. The resulting multimodal feature set is subsequently used as inputs to CML algorithms (e.g. Naive Bayes, rpart, Random Forest), thereby improving interpretability and transparency compared to end-to-end DL approaches (Balek *et al*. 2025).

Table 2 presents a comparative analysis of ten representative papers on clinical outcome prediction using EHR data. These papers were chosen based on their thematic relevance to the proposed framework along with at least one of the following criteria: use of MIMIC data, prediction of ICU mortality or readmission as the target outcome, inclusion of clinical notes, combination of severity scores, or implementation of multimodal and hybrid architectures. Each entry in the table contains the prediction task, the data source, the input types, the prediction method, the interpretability mechanism, and the main qualitative difference from the proposed approach.

**Table 2:** Comparative overview of related work on clinical outcome prediction from EHR data.

Work	Task and outcome	Data modalities	Method	Interpretability	Key limitation vs. proposed
Yang *et al.* (2023)	90-day ICU mortality	Struct. Only	9 CML models; best XGB	SHAP	No NLP, no severity score integration
Hou *et al.* (2020)	30-day mortality, sepsis	Struct. + SAPS-II (comparison)	XGB vs. LR/SAPS-II	Nomogram	Struct. baseline only; no text
Hempel *et al.* (2023)	ICU LOS	Struct. Only	CML models	–	Different outcome; no text or scores
Wu *et al.* (2024)	HF ICU mortality; 3/30/365d	Struct. + discharge notes	BERTopic + XGB/LightGBM	Gini, topic keywords	Disease-specific (HF); no SOFA/SAPS/LODS
Zhang *et al.* (2024)	ED severity assessment	Struct. + unstructured	Multimodal ML	–	Different setting (ED); no ICU scores
Chiu *et al.* (2024)	ICU readmission, 30d	Struct. + discharge notes	BERTopic + LSTM + CML	Topic labels	Readmission, not mortality; no severity scores
Soenksen *et al.* (2022)	12 tasks incl. 48 h mortality	Tabular, time-series, text, images	HAIM; DL framework	Shapley per modality	Complex DL; limited clinical transparency
Lyu *et al.* (2023)	In-hospital mortality	Struct. EHR + clinical notes	Multimodal Transformer + ClinicalBERT	Integrated gradients	End-to-end DL; lower interpretability
Grnarova *et al.* (2016)	Mortality from notes	Text only	CNN/doc. embeddings	Limited	Text-only; no structured or score fusion
Gana *et al.* (2025)	Mortality from notes	Text only	BERT/BlueBERT/TF-IDF	Limited	Text-only; no struct. or score fusion
**Proposed**	**Short-term ICU mortality**	**Struct. + SOFA/SAPS II/LODS/CCI + discharge notes**	**BERT [CLS]** → **Autoencoder** → ***K*-Means** → **cluster ID + CML (RF, NB, GLM, RPART, GBM)**	**SHAP; semantic clusters**	**–**

Abbreviations: Struct., structured data; CML, classical machine learning; DL, deep learning; LLM, large language model; RF, Random Forest; XGB, XGBoost; LSTM, Long Short-Term Memory; GLM, Generalised Linear Model; NB, Naive Bayes; HF, heart failure; ICU, Intensive Care Unit; LOS, length of stay.

Many studies only consider structured clinical variables, ignoring unstructured free-text documentation. Yang *et al*. (2023) and Hou *et al*. (2020) show effective performance with gradient-boosted models and SHAP values for interpretability, but their predictive capability is limited by the tabular nature of the data. Works that use clinical text as an additional modality, notably by Wu *et al*. (2024) and Chiu *et al*. (2024), achieve large gains over models trained only on structured data, highlighting the benefits of unstructured clinical language. However, these contributions remain anchored to disease-specific or outcome-specific contexts. The multimodal transformer of Lyu *et al*. (2023) and the HAIM framework of Soenksen *et al*. (2022) exemplify the potential for deep end-to-end fusion across various modalities, however their reliance on complex neural architecture is a significant trade-off between performance and interpretability. The text-only models of Grnarova *et al.* (2016) and Gana *et al*. (2025) highlight the predictive power of clinical notes in isolation, but disregard the structured and score-based clinical signal completely.

The proposed framework has a unique methodological advantage in this landscape. Rather than attempting an end-to-end deep learning fusion, it breaks down the multimodal fusion problem into two intuitive steps: (1**)** compressing and discretizing dense BERT embeddings of discharge summaries into one categorical semantic feature using K−means clustering, and (2**)** concatenating the semantic feature with structured clinical variables and explicitly computed severity scores into a single tabular input for classical machine learning classifiers. This design maintains the clinical semantics of validated prognostic tools, converts opaque continuous embeddings into categorical features that are auditable and amenable to SHAP analysis, and is compatible with interpretable and regulatory-friendly algorithms.

## 4 Material and methods

**Figure 1:**
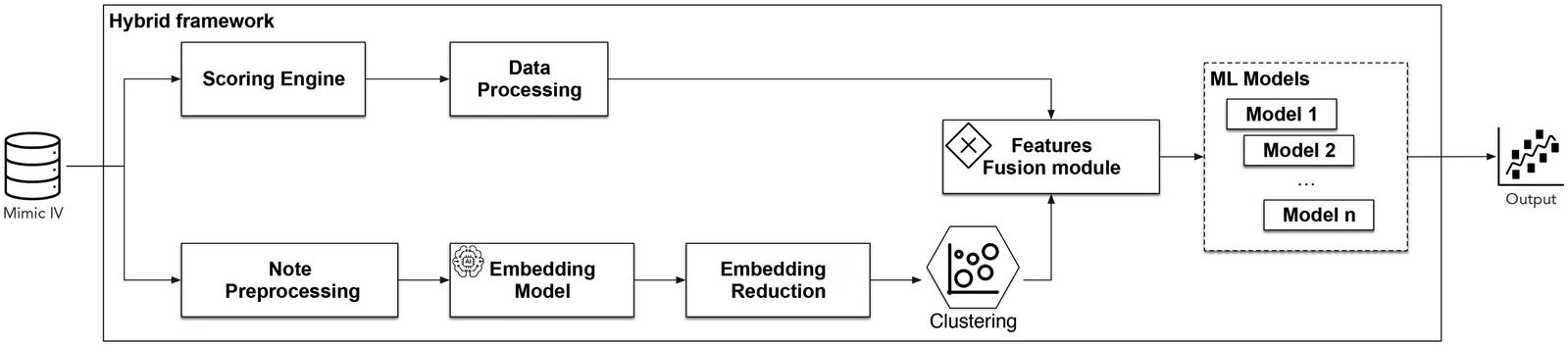
Architecture of the proposed framework. The pipeline integrates two parallel branches: (i) the tabular data branch, which processes tabular features and derives clinical scores; and (ii) a clinical notes branch, which extracts semantic representations from discharge summaries via sentence embeddings, dimensionality reduction, and unsupervised clustering. The outputs of the two branches are merged in the feature-fusion module and are then used to train machine learning classifiers.

Figure 1 shows the architecture of the proposed hybrid framework designed to integrate multimodal clinical inputs. The workflow begins with the extraction of relevant data from the MIMIC-IV database, which are then processed through dedicated modules. Following data extraction, the framework splits into two separate branches, each responsible for processing the two different modalities of clinical information: the *Clinical Notes Branch* and the *Tabular Data Branch*. Each branch includes a dedicated preprocessing pipeline tailored to the characteristics of its input. The outputs from both branches are then fed into the *Features Fusion* module, which concatenates the structured clinical variables with text embeddings generated by a BERT-based model and subsequently reduces dimensionality. This module assigns each clinical note to a semantic cluster, and the corresponding *Cluster ID* is encoded as a categorical variable and appended to the table of structured clinical variables. The resulting unified table of features serves as input to the final stage of the framework, which consists of a set of ML models. Since all ML models are evaluated on the same input features, this design enables a direct and fair comparison of predictive performance across algorithms. The two branches are described as follows:

### 4.1 Clinical notes branch

**Figure 2:**
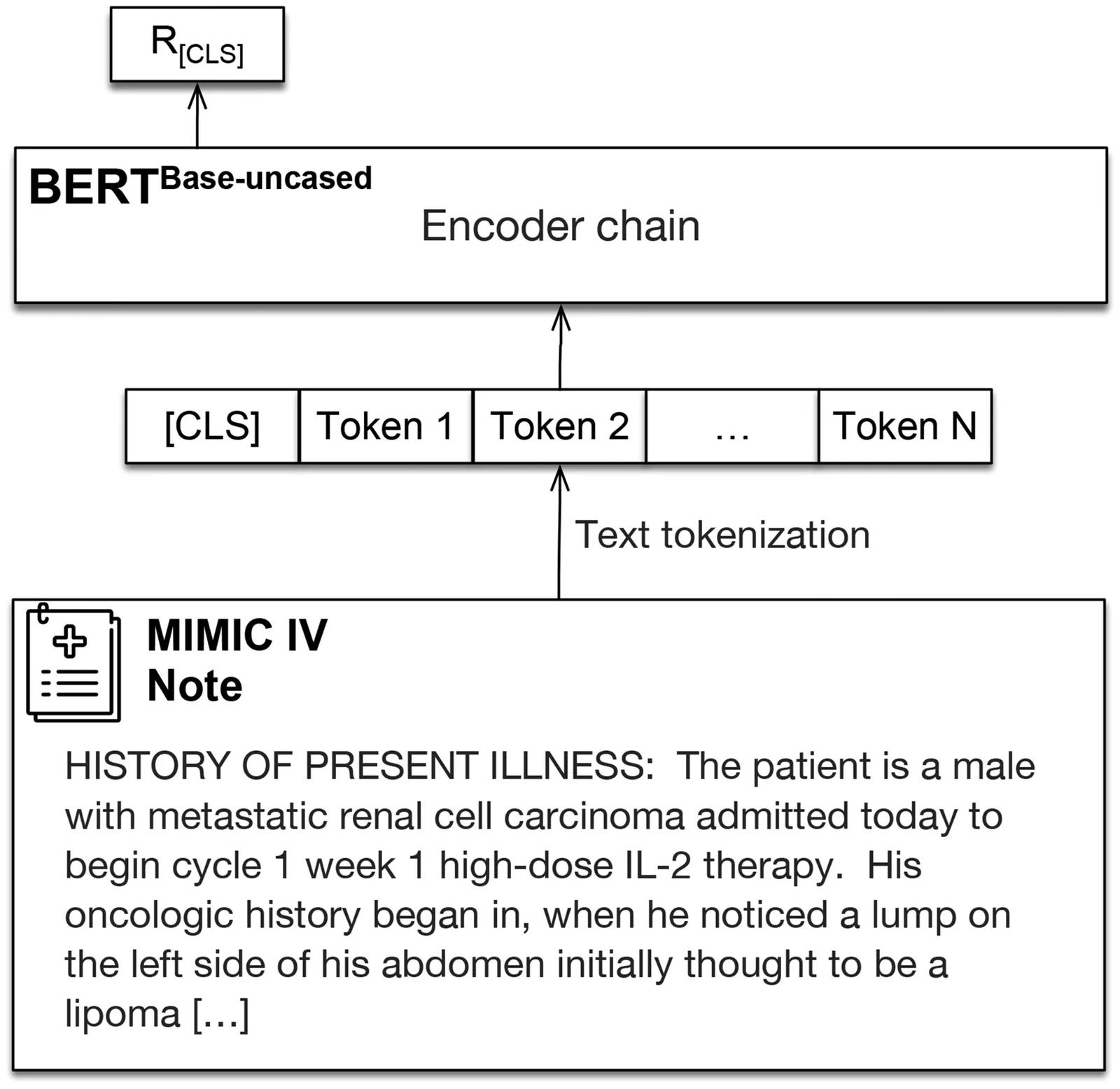
Architecture of $\mathrm{BER}T^{\mathrm{Base}-\mathrm{uncased}}$ applied to the processing of clinical notes. Each note, after tokenization, passes through 12 encoder layers, producing contextualized representations of the clinical note as a vector of size 768.

This branch unfolds through the following key stages. *Note Preprocessing* reduces noise in the data, ensuring that the subsequent BERT tokenization process produces meaningful and consistent input representations. The raw clinical notes are cleaned and standardized by removing irrelevant characters, extraneous punctuation, and artifacts introduced during data entry or de-identification procedures. In the *Embedding Model* stage, preprocessed clinical notes are fed into a BERT-based model to generate contextualized token embeddings, as shown in Fig. 2. For each input sequence, the embedding corresponding to the special [CLS] token is extracted and used as a fixed-length vector representation of the entire note, capturing the global contextual features of the clinical condition. The resulting [CLS] embeddings then undergo *Embedding Reduction*, where dimensionality is reduced to preserve essential semantic features while enabling more efficient downstream processing and clustering. Finally, in the *Clustering* stage, the reduced embeddings are grouped using a clustering algorithm. The optimal number of clusters is determined using the Davies-Bouldin Index (DBI) (Rubiños *et al*. 2024), an internal evaluation metric that quantifies the trade-off between intra-cluster compactness and inter-cluster separation, defined as:


(5) $$\text{DBI}=\frac{1}{k}\sum_{i=1}^{k}\underset{j\neq i}{\text{max}}\left(\frac{\sigma_{i}+\sigma_{j}}{d_{ij}}\right)$$

where *k* represents the number of clusters, $\sigma_{i}$ and $\sigma_{j}$ are the average distances between each point of cluster *i* or *j* from their respective centroids, and $d_{ij}$ is the distance between the centroids of clusters *i* and *j*. Lower DBI values indicate more compact and well-separated clusters. Each cluster is assigned a unique *Cluster ID*, and each clinical note is mapped to one of the resulting semantic clusters, enabling a structured analysis of clinically related semantic patterns within the dataset.

### 4.2 Tabular data branch

This branch unfolds through the following key modules:

•  *Clinical Scores engine*: Clinical scores (e.g. SOFA, SAPS II) are computed based on primary input variables derived from raw clinical measurements, including blood pressure, respiratory rate, and laboratory test results. Each score is computed according to its published criteria, applying rule-based logic and clinically validated thresholds. These calculations are typically performed within predefined time windows, such as the first 24 hours of ICU admission, to ensure consistency and clinical relevance.
•  *Data Preprocessing*: This module addresses several issues in raw data, including missing values, formatting inconsistencies, and outliers that may compromise the reliability of the downstream analysis (Settino *et al*. 2025). The main steps in the preprocessing pipeline are:
–  *Feature Extraction* involves selecting and combining variables from raw data to reduce dimensionality, improve model performance, and simplify the learning process;
–  *Data cleaning* involves handling missing values, removing duplicates, standardizing data formats, and correcting errors and inconsistencies in the data to ensure consistency and reliability for downstream analysis;
–  *Handling outliers* involves detecting data points that significantly deviate from the expected distribution of a variable and applying appropriate methods to mitigate their potential impact on the analysis and predictive modeling.
–  *Data Normalizing* preserves the overall distribution of the original dataset and involves scaling features with different units and magnitudes to a common range to ensure that all values are handled consistently;
–  *Class Imbalance Handling* involves addressing the problem of unequal class representation in a dataset that arises when one class has significantly more observations than another. Such an imbalance can lead ML models to become biased toward the majority class, resulting in poor performance on the minority class. A metric used to measure the degree of imbalance in the dataset is the Imbalance Ratio (IR), defined as:
  (6) $$IR=\frac{N_{\text{major}}}{N_{\text{minor}}},$$

where $N_{\text{major}}$ and $N_{\text{minor}}$ are the numbers of instances in the majority and minority classes, respectively. An IR value of 1 indicates a perfectly balanced dataset with equal class representation, while higher IR values reflect class imbalance.

### 4.3 MIMIC-IV dataset and study design

The study included approximately 1 80 000 ICU admission records extracted from the MIMIC-IV database between 2008 and 2019, corresponding to approximately 50 000 unique patients identified through *subject_id*. Since patients could be admitted to the ICU multiple times, each hospital admission identified through *hadm_id* was treated as an independent observation.

Structured features are classified into *primary* and *derived* variables, depending on whether they are directly collected from EHRs or computed by combining multiple raw inputs from EHRs.

*Primary variables* were further grouped into five categories: (i) *Demographic data* include basic patient information such as age, sex, and ethnicity; (ii) *Vital signs data* consist of key physiological measurements, including heart rate, respiratory rate, blood pressure, and body temperature, that provide immediate insight into the patient’s clinical status; (iii) *Laboratory parameters values* cover results from blood chemistry and hematology analyses, such as white blood cell count, hemoglobin levels, and serum creatinine, which reflect the patient’s physiological and pathological conditions; (iv) *Administrative data* refer to institutional information such as length of hospital stay and the number of ICU admissions per patient; and (v) *Pharmaceutical data* comprise information on medications administered during hospitalization, reflecting the therapeutic management and clinical treatment strategies implemented during ICU care. *Derived variables* comprise clinical scores, as they were obtained by aggregating ple primary variables into a single summary score (see Subsection 2.3).

## 5 Experimental setup

This experimental setup assesses whether incorporating semantic features extracted from unstructured clinical notes improves short-term ICU mortality prediction compared with ML models trained solely on structured data. Short-term mortality in ICU patients was defined as the outcome of interest and used as the target variable for model training and evaluation.

The proposed framework follows a retrospective admission-level design in which all structured variables and discharge clinical notes associated with each hospital admission were included in the analysis. Therefore, the study was not based on a predefined early observation window. However, some clinical severity scores (e.g. SOFA, SAPS II, and LODS) were computed according to their standard clinical definitions using physiological measurements collected during the first 24 hours of ICU admission.

Each hospital admission identified through *hadm_id* was treated as an independent observation. Therefore, no averaging or aggregation of structured variables or clinical notes across multiple admissions belonging to the same patient was performed. This design avoids the potential temporal leakage and clinical inconsistencies that could arise from combining information across unrelated hospitalizations separated by long time intervals.

Likewise, total ICU length of stay, i.e. *total_icu_los* was excluded from the predictive feature set to reduce the risk of temporal leakage associated with hospitalization duration.

In the experimental setup, the *BERT-base-uncased* model is employed to transform unstructured text into meaningful semantic representations that capture latent patterns within clinical notes.

### 5.1 Experimental dataset

The study cohort was obtained by stratified random partitioning of the original patient-level dataset into training and test sets, while preserving the class distribution across the two subsets. The main demographic and clinical characteristics of the resulting cohorts are reported in Table 3, where statistics were computed after the train/test split and before any data balancing, feature selection, or preprocessing procedures.

**Table 3:** Patient characteristics in the training and test sets.

Variable	Training set	Test set
Cohort size	700	300
Age	66.02 (54.68–76.32)	65.05 (55.63–75.47)
Total ICU LOS	3.36 (1.53–8.72)	3.65 (1.71–9.93)
Heart rate, mean	82.19 (74.09–92.21)	83.96 (73.28–97.91)
SBP, mean	116.96 (107.16–131.3)	115.23 (106.25–125.82)
DBP, mean	62.75 (55.35–71)	63.6 (56.25–68.43)
MBP, mean	77.93 (70.56–85.99)	77.6 (71.19–82.46)
Respiratory rate, mean	17.9 (16.06–20.67)	18.37 (16.11–21.33)
Temperature, mean	36.83 (36.65–37.1)	36.8 (36.6–37.04)
SpO_2_, mean	97.11 (95.75–98.34)	97 (95.56–98.5)
Glucose, mean	129 (110.67–152.8)	127.5 (109.8–151.5)
Female	338 (48.3%)	143 (47.7%)
Male	362 (51.7%)	157 (52.3%)
Alive	653 (93.3%)	276 (92%)
Dead	47 (6.7%)	24 (8%)

Continuous variables are reported as median (IQR), whereas categorical variables are reported as *n* (%).

The dataset used in this study was organized around hospital admissions identified by *hadm_id*. Multiple records could be associated with the same admission due to the integration of heterogeneous structured and unstructured clinical data sources.

A complete summary of the variables used in this study is provided in Table 4. Additional details and definitions can be found in the official MIMIC-IV documentation (https://mimic.mit.edu). Due to limitations in memory and computational resources of the experimental environment used for the experiments (see the following Section 5.2), the analysis was conducted on a representative subset comprising 60% of the full dataset. This approach is based on the assumption that ML models do not necessarily require all available data, but can instead benefit most from training on informative and representative samples selected according to their relevance to the target task (Kossen *et al*. 2021).

**Table 4:** Representativeness check comparing the full dataset with a 60% subset.

Variable	KS *p*-value	*t*-test *p*-value	sig. KS/*t*-test
**Scores**			
CCI	1.00	0.45	_**✓**_/_**✓**_
avg_lods	1.00	0.63	_**✓**_/_**✓**_
avg_sapsii	1.00	0.71	_**✓**_/_**✓**_
avg_sofa_24hours	0.96	0.39	_**✓**_/_**✓**_
**Administrative**			
icu_stay_count	1.00	0.89	_**✓**_/_**✓**_
total_icu_los	0.98	0.78	_**✓**_/_**✓**_
**Demographic**			
age	1.00	0.92	_**✓**_/_**✓**_
**Vitals**			
heart_rate_mean	1.00	0.70	_**✓**_/_**✓**_
sbp_mean	0.92	0.57	_**✓**_/_**✓**_
dbp_mean	1.00	0.81	_**✓**_/_**✓**_
mbp_mean	1.00	0.55	_**✓**_/_**✓**_
resp_rate_mean	0.96	0.76	_**✓**_/_**✓**_
spo2_mean	0.96	0.77	_**✓**_/_**✓**_
**Laboratory**			
hematocrit	0.98	0.61	_**✓**_/_**✓**_
hemoglobin	0.87	0.61	_**✓**_/_**✓**_
mch	1.00	0.87	_**✓**_/_**✓**_
mchc	1.00	0.97	_**✓**_/_**✓**_
mcv	1.00	0.83	_**✓**_/_**✓**_
platelet	1.00	0.82	_**✓**_/_**✓**_
rbc	0.68	0.75	_**✓**_/_**✓**_
rdw	1.00	1.00	_**✓**_/_**✓**_
wbc	1.00	0.31	_**✓**_/_**✓**_

KS, Kolmogorov–Smirnov test.

Reducing the dataset size enabled more efficient model training and evaluation while preserving the statistical representativeness of the original dataset and maintaining the reliability of the results. Following the method proposed in the work (Ortell *et al*. 2019), a representative subset of the original data was selected from multiple candidate subsets generated through by random sampling. The Kolmogorov-Smirnov (KS) test was used to assess distributional differences, while the Student’s *t*-test was used to evaluate differences in means (Al-Kassab 2022, Cardoso and Galeno 2023). Only subsets that showed no statistically significant differences (P>0.05) from the original dataset across all tested variables were retained, ensuring that the final subset accurately reflected the characteristics of the full cohort. One of the retained representative subsets was then randomly selected for subsequent analysis.

Table 4 reports the results of comparing the full dataset with the representative subset using the statistical test. For each variable, the corresponding *P*-value is reported together with an indication of statistical significance. The checkmark (_**✓**_) denotes no significant difference (P≥0.05), indicating that the subset is representative, while the exclamation mark (**!**) denotes a significant difference (P<0.05), suggesting potential bias. The results confirm that the subset used in this study reliably represents the full dataset.

Table 5 reports the cohort selection flow diagram with counts reported at each exclusion step adopted to construct the proposed multimodal framework. Admissions without available discharge summaries were excluded because discharge notes were required for the clinical notes branch of the framework.

**Table 5:** Cohort selection flow diagram with counts reported at each exclusion step adopted to construct the proposed multimodal framework.

Cohort selection step	Records	Admissions	Patients
Initial dataset extracted from MIMIC-IV	1000	954	926
Excluded admissions without available discharge summaries	−36	−36	−32
Final multimodal analytical cohort	964	918	894

**Figure 3:**
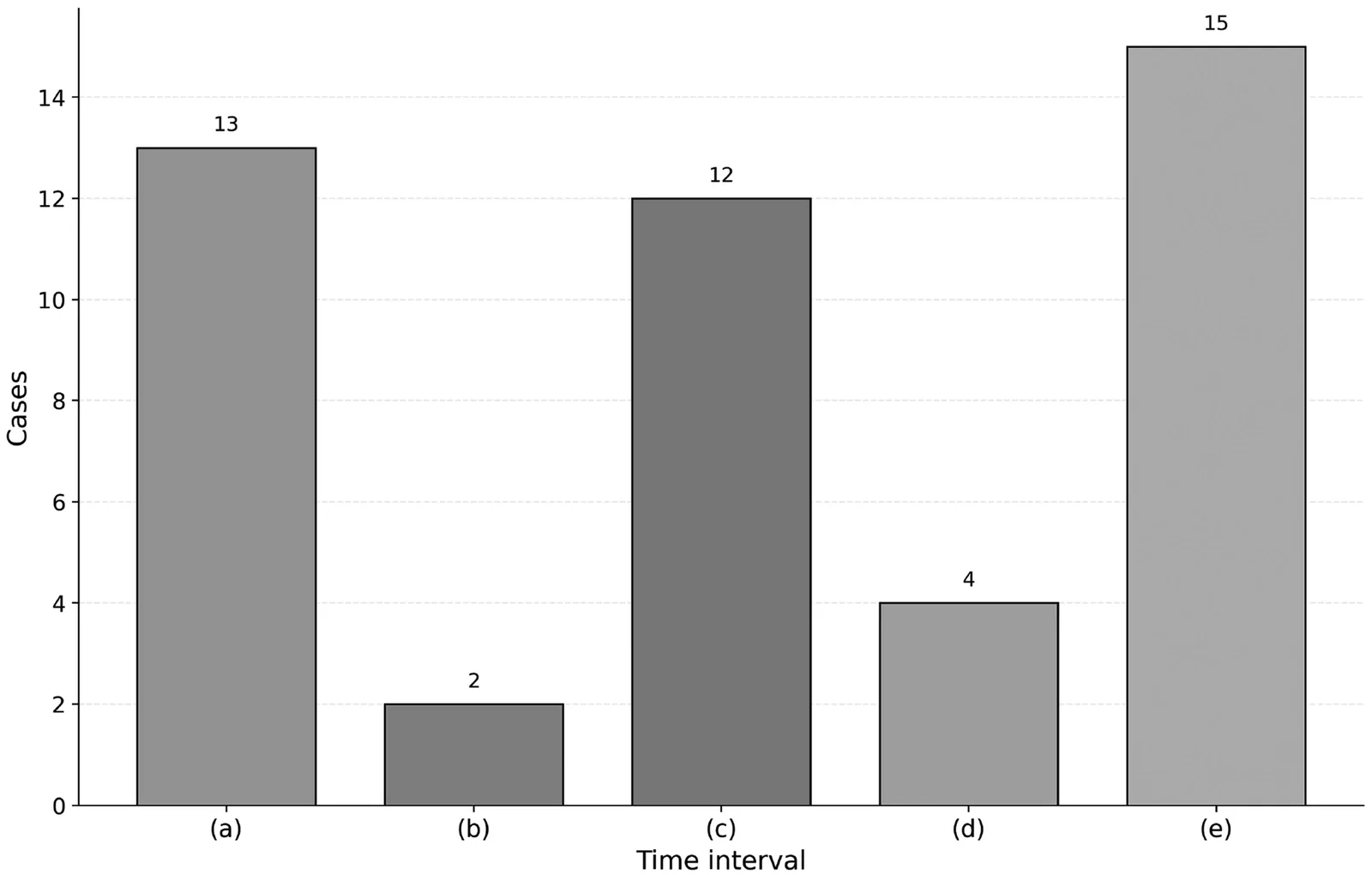
Temporal distribution of mortality events in the short-term prediction window: (a) during hospital stay, (b) 1 day after discharge, (c) 2–7 days after discharge, (d) 8–15 days after discharge, and (e) 16–30 days after discharge.

Figure 3 illustrates the temporal distribution of mortality events considered in the definition of the short-term mortality outcome. Mortality events were distributed across different time intervals, including during hospitalization and within 30 days after discharge, highlighting the temporal heterogeneity of the outcome and supporting the clinical relevance of the adopted prediction window.

### 5.2 Computational environment

The computational experiments were conducted on a server running Ubuntu 22.04.5 LTS with a 13th Gen Intel Core i9-13900KF processor. The system is equipped with 32 logical cores and supports both 32-bit and 64-bit operation modes. It has 62 GiB of RAM, with approximately 44 GiB available after system processes. The CPU operates at a base frequency of 800 MHz, with a maximum boost up to 6.0 GHz, and includes a 36 MiB L3 cache. The system architecture is x86_64. The proposed framework (see Fig. 1) was implemented in R (ver. 4.4.2) and Python (ver. 3.11). Specifically, the “Tabular Data Branch” was developed in R, leveraging functions from the Caret package to preprocess structured data, build, and train ML models, whereas the “Clinical Notes Branch” was implemented in Python using the packages: *pandas* for data handling, *torch* for deep learning operations, and *transformers* for embedding clinical notes.

The confusion matrix and other metrics, including accuracy (Acc), sensitivity (Sens), specificity (Spec), precision (Prec), recall (Rec), F1 score (F1), Cohen’s Kappa (Kappa) and area under the ROC curve (AUC) were recovered to provide a comprehensive evaluation of model performance. In addition, to graphically represent the performance of a classification model, pROC package functions are used to perform the ROC curves (receiver operating characteristic). The publicly available code from MIT-LCP was adapted and used to calculate clinical scores of the primary variables extracted from MIMIC-IV. Custom modifications were made to align with the requirements of this study (MIT Laboratory for Computational Physiology 2019).

### 5.3 Data extraction

Raw data were extracted from the MIMIC-IV Database using Structured Query Language (SQL) queries within the PostgreSQL environment. The dataset consists of 1.507423 rows and 41 columns. Feature selection was guided by clinical expertise to capture both initial physiological status and treatment progression. Following this process, 22 clinically relevant features were retained for the analysis.

The dataset was organized at the hospital-admission level using *hadm_id* as the admission identifier, while patients were uniquely identified through *subject_id*. This admission-level design was adopted to avoid combining potentially unrelated clinical episodes occurring across different hospitalizations separated by long temporal intervals. Consequently, multiple hospital admissions could be associated with the same patient. Clinical discharge notes were extracted exclusively from the *mimiciv_note.discharge* table and linked to structured clinical data using both *subject_id* and *hadm_id*. Therefore, only discharge summaries were included in the proposed framework, whereas other note categories available in MIMIC-IV, such as radiology reports, nursing notes, and physician progress notes, were not considered in this study. Since multiple admissions could belong to the same patient, each discharge note was independently linked to its corresponding hospital admission through the associated *hadm_id*. No concatenation of notes and no averaging of embeddings across admissions were performed. Accordingly, the prediction task was formulated at the hospital-admission level, where admissions and their associated discharge notes were treated as independent observations within a retrospective multimodal framework rather than as an early time-dependent prediction task. The extracted notes were subsequently processed through the clinical notes branch to generate contextual semantic representations integrated with structured clinical variables within the multimodal framework.

The target variable (alive) was not directly available in the MIMIC-IV dataset and was therefore derived. Specifically, alive was constructed by combining the recorded date of death (dod) from the patients table with the admission and discharge timestamps (admittime, dischtime) from the admissions table.

The outcome variable was defined as non-survival (alive = 0) when death occurred either during the hospital admission or within 30 days following hospital discharge. All remaining cases, including patients without a recorded date of death within this time window, were labeled as alive (alive = 1). The date-of-death field (*dod*) was used exclusively to derive the outcome variable and was not retained in the predictive feature matrix or in the subsequent SHAP interpretability analysis.

### 5.4 Clinical notes branch

The clinical notes branch was designed to convert free-text discharge summaries into an explicit semantic feature that could be integrated with structured clinical variables. To avoid information leakage, the stratified train-test split was performed before fitting any trainable component of this branch. Consequently, dimensionality reduction and clustering were learned only from the training notes, while the notes in the held-out test set were transformed and assigned to clusters using the fitted models.

The branch was implemented through the following steps:

- *Note Preprocessing*: clinical notes were standardized before tokenization by converting text to lowercase, in accordance with the *BERT-base-uncased* model, and by removing textual artifacts introduced during data entry or de-identification. In particular, tokens containing extended underscores, excessive punctuation, non-ASCII characters, and special symbols *(*e.g. #, (), %, *⁢*, £,* !*) were filtered to reduce noise and minimize fragmentation within the token vocabulary.
- *Embedding Extraction*: after preprocessing, each clinical note is tokenized and truncated to a maximum length of 512 tokens, adhering to the input constraints of $\mathrm{BER}T^{\mathrm{Base}-\mathrm{uncased}}$. It generates contextualized embeddings from which the [CLS] token representation (a 768-dimensional vector) is extracted.
- *Embedding Reduction*: the high-dimensional [CLS] embeddings are compressed from 768 dimensions to 128 dimensions using a fully connected autoencoder consisting of a symmetric encoder-decoder architecture. The encoder maps the input through a 256-dimensional linear layer, followed by ReLU activation, and then into the latent dimension. The decoder mirrors this process to reconstruct the input. The reduction process creates an information bottleneck in the low-dimensional latent space, which preserves relevant features while reducing noise, redundancies, and spurious variations inherent in high-dimensional BERT embeddings, such as overfitting to artifacts specific to the dataset (Wang *et al*. 2016).
- *Clustering*: the reduced 128-dimensional training embeddings were clustered using the *k-means* algorithm with cosine distance as the similarity measure. Four distinct clusters were identified by using the DBI metric (see Equation 5), and *k-means* centroids were estimated only from the reduced training embeddings. Among these, three exhibited coherent and clinically meaningful themes, while one (Cluster 0) represents notes with minimal or no informative content, possibly due to missing, incomplete, or non-descriptive documentation. Each clinical note was assigned to one of the identified clusters according to its embedding, and the corresponding *Cluster ID* was encoded as a categorical feature. Table 6 provides a description of the thematic content of the meaningful clusters. Together, these clusters provide a structured view of thematically distinct areas of clinical care.

**Table 6:** Overview of semantic clusters identified through *k*-means on clinical note embeddings.

Cluster ID	*N*	Mortality (%)	Likely theme	Interpretation summary
0	102	6.86	Non-informative or non-specific documentation	After keyword analysis, no consistent clinical theme emerged. Predominant terms are mostly standardized formulations, medical prescriptions and documentation, rather than clinically specific content.
1	208	7.21	Mixed acute care and procedural documentation	Includes broad clinical and procedural language, with references to surgery, incision, wound, abdominal, CT, and IV care.
2	221	8.14	Acute cardiopulmonary/diagnostic documentation	Includes terms such as chest, CT, pulmonary, ventricular, and negative, suggesting acute diagnostic and cardiopulmonary contexts.
3	387	4.39	Cardiothoracic and valve-related documentation	Contains terms such as chest, valve, aortic, and related procedural vocabulary.

Cluster size and mortality prevalence were computed on the original multimodal cohort of admissions before class balancing.

### 5.5 Tabular data branch

This branch was implemented as follows. The *Clinical Scores engine* is a dedicated module designed to compute clinical scores by aggregating relevant physiological and clinical variables according to established scoring guidelines and formulas. The resulting scores, represented in tabular form, are appended to the table of primary structured clinical variables and jointly used as input to the subsequent *Data Processing* pipeline, which includes several processing steps. First, variables with more than 60% missing values are removed to balance data completeness with information retention. The remaining missing values are then imputed using the *median* of each variable, calculated only from the training set. This approach prevents data leakage and ensures a fair evaluation of the held-out test set. Next, variables with zero variance, i.e. having the same value across all samples, are discarded, as they do not contribute to the predictive power of the model. To further reduce redundancy and multicollinearity, a correlation matrix is computed on numeric features from the training set, and variables with a Pearson correlation coefficient above 0.9 are eliminated. Subsequently, all features are standardized using *z*-score normalization, subtracting the mean and dividing by the standard deviation of the training set; the same transformation is applied to the held-out test set to ensure consistency. Finally, to address class imbalance (the initial Imbalance Ratio was approximately 4.77), the *ROSE* (Random OverSampling Examples) technique was applied to the training set. As a result, the class distribution becomes approximately balanced, with a resulting IMR close to 1.

Before model fitting, patient and admission identifiers (*subject_id*, *hadm_id*), target-defining variables (e.g. *dod*), raw text fields and note metadata (*text*, *note_type*), timestamp variables (e.g. *lab_charttime*), and *total_icu_los* were excluded from the predictor set. In the multimodal setting, only the semantic Cluster ID derived from the clinical notes branch was preserved as a note-based feature and encoded as a categorical variable.

### 5.6 Performance comparison

Initially, five ML classifiers, each representing a major algorithmic family, were trained solely on primary structured data (see Subsection 4.3) to serve as baseline references.

**Figure 4:**
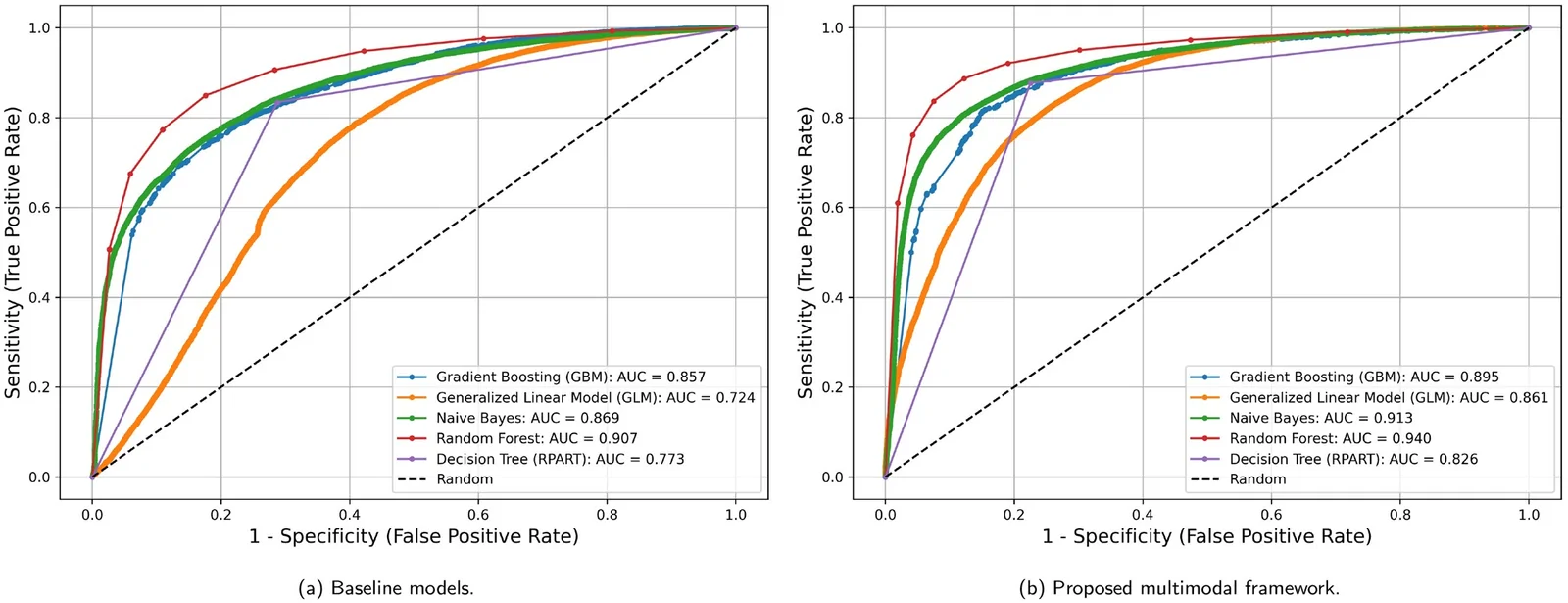
Comparison of AUC-ROC curves for the baseline models and the proposed hybrid model. Panel (a) presents the AUC-ROC curves of the baseline models, which rely only on primary structured variables. Panel (b) shows the performance of the proposed hybrid framework, which incorporates primary structured variables, derived structured clinical scores, and unstructured clinical notes.

The predictive performance of the baseline ML classifiers is summarized in the upper section of the Table 7, with corresponding ROC curves shown in Fig. 4a**)**.

**Table 7:** Comparative performance of ML models for 30-day mortality prediction under baseline and multimodal settings.

Setting	Model	Acc	Sens	Spec	Prec	Rec	F1	Kappa	AUC
**Baseline**	**RF**	**0.840**	0.850	**0.824**	**0.892**	0.850	**0.870**	**0.662**	**0.907**
	NB	0.792	0.870	0.658	0.813	0.870	0.840	0.541	0.869
	GBM	0.757	**0.958**	0.413	0.736	**0.958**	0.833	0.416	0.857
	RPART	0.789	0.834	0.713	0.832	0.834	0.833	0.547	0.773
	GLM	0.729	0.906	0.426	0.730	0.906	0.808	0.363	0.724
**Multimodal**	**RF**	**0.884**	0.887	**0.878**	**0.925**	0.887	**0.906**	**0.754**	**0.940**
	NB	0.841	0.893	0.753	0.861	0.893	0.876	0.654	0.913
	GBM	0.817	**0.943**	0.601	0.802	**0.943**	0.866	0.580	0.895
	GLM	0.806	0.898	0.648	0.814	0.898	0.854	0.568	0.861
	RPART	0.839	0.877	0.775	0.869	0.877	0.873	0.654	0.826

The baseline setting includes only structured primary variables, while the multimodal setting integrates structured primary variables, clinical scores, and unstructured clinical notes.Note: Bold values indicate the best performance for each metric within a given setting, while the bolded model name (RF) indicates the overall best-performing model, as determined by the highest AUC.

Subsequently, the same classifiers were independently retrained on multimodal data using the proposed hybrid framework, which integrates structured variables with semantic features extracted from unstructured clinical notes. The predictive performance of the hybrid framework is summarized the lower section of the Table 7, with corresponding ROC curves shown in Fig. 4b**)**.

The positive class used during model evaluation corresponded to the “alive” class. Therefore, sensitivity, recall, precision, and F1-score refer to the correct identification of surviving patients.

All machine learning models were trained using features derived from a stratified split between the training set and the test set in a 66%/34% ratio, with stratification based on the “alive” feature to preserve class balance. The held-out test set was kept strictly separate and used exclusively for final evaluation, ensuring an unbiased estimate of generalization performance. Hyperparameter tuning was conducted exclusively on the training set via *k*-fold stratified cross-validation, with k=5 and 10 repetitions, following standard best practices in supervised machine learning (Varma and Simon 2006). Models were trained using the default parameter settings of the caret package implementation in R (https://topepo.github.io/caret/).

All ML models show notable improvements across key metrics when clinical scores and clinical notes are incorporated into input features. Even weaker-performing models in the baseline setting (e.g. GLM, GBM) benefit significantly from the multimodal integration. Among all classifiers, Random Forest demonstrates the highest discriminative ability, achieving an AUC of 0.940. Notable improvements include increased specificity (0.824 → 0.878), precision (0.857 → 0.901), and Cohen’s Kappa (0.662 → 0.754), indicating enhanced reliability and a reduction in false positives.

### 5.7 SHAP-based interpretability analysis

To analyze the interpretation of the final Random Forest model, SHAP values were computed on the held-out test set using the final preprocessed feature matrices for both the baseline and multimodal settings (Lundberg and Lee 2017). The overall relevance of the features was summarized using the absolute mean SHAP value, which allowed for a comparison of each predictor’s contribution on a standardized scale. In the multimodal model, the semantic cluster ID, obtained from the embeddings of the clinical notes, was encoded in one-hot format, and its total contribution was determined by summing the absolute mean SHAP values of the corresponding indicator variables *note_cluster* and comparing this total with the overall contribution of the structured clinical predictors.

**Figure 5:**
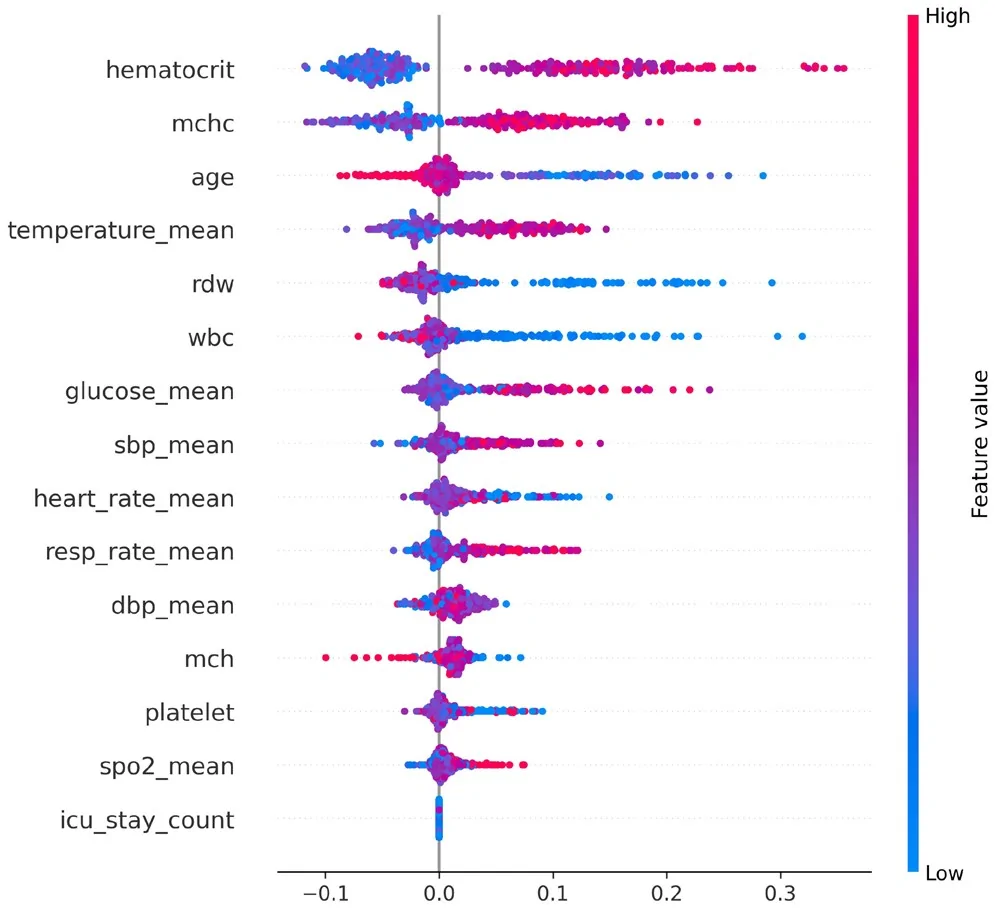
SHAP summary for the baseline Random Forest model. Features are ranked by mean absolute SHAP value computed on the held-out test set, reflecting their relative global importance.

**Figure 6:**
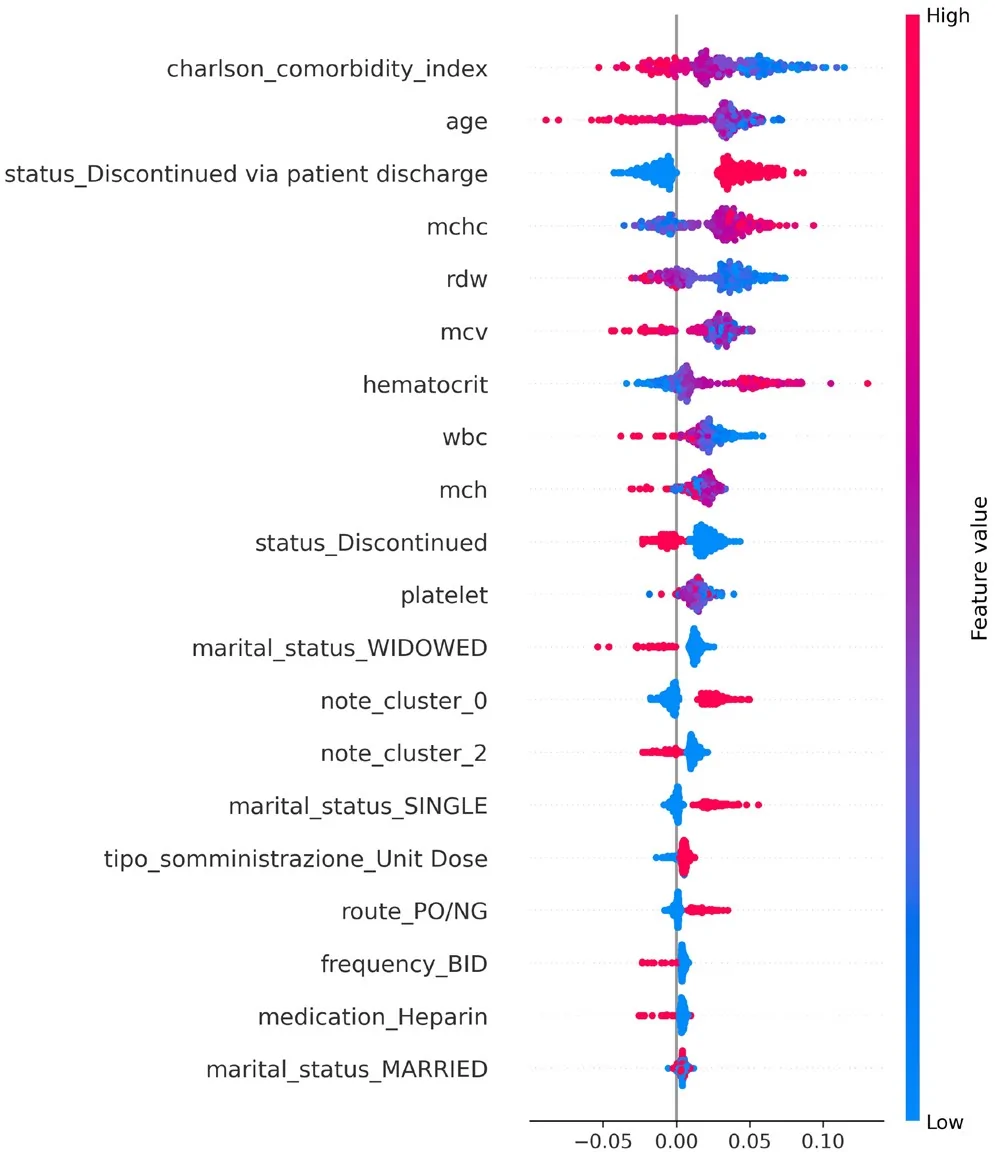
SHAP summary for the multimodal Random Forest model. Features are ranked by mean absolute SHAP value computed on the held-out test set, reflecting their relative global importance. The semantic Cluster ID derived from clinical-note embeddings is represented by the one-hot-encoded *note_cluster* indicator variables, shown alongside the structured clinical predictors.

The SHAP analysis showed that structured clinical variables remained the dominant source of predictive signal in the final Random Forest model, while the Cluster ID provided an additional semantic contribution extracted from discharge summaries. Specifically, the Cluster ID accounted for 5.00% of the total mean absolute SHAP importance, whereas the remaining contribution was attributable to structured predictors. The baseline and multimodal SHAP summary plots are reported in Figs 5 and 6, respectively.

## 6 Discussion and conclusions

This study proposes a novel multimodal framework for retrospective short-term mortality prediction in ICU patients through the integration of structured clinical variables, validated clinical scores, and semantic information extracted from unstructured discharge summaries. The proposed framework improves the characterization of patient clinical profiles and highlights the potential of multimodal approaches to support future prospective early-warning systems for ICU mortality risk assessment, enabling earlier identification of high-risk patients and more timely clinical interventions.

Data were obtained from the MIMIC-IV database. A BERT-based model was used to generate contextual embeddings from clinical notes, which were then clustered using K-Means into four semantic groups. Each note was labeled with a cluster ID, later encoded as a categorical feature.

This semantic feature gathered from the clusters was integrated with structured clinical variables using the Feature Fusion module, generating a unified feature matrix. Five ML classifiers, i.e. GBM, RF, RPART, GLM, NB were trained on this enriched input.

The results obtained showed that the proposed hybrid approach effectively uses complementary information from both structured and unstructured data modalities, leading to improved predictive performance compared to baseline ML models trained exclusively on structured data.

Among all classifiers, RF demonstrates the highest discriminative ability, achieving an AUC of 0.940 in the hybrid setting, outperforming all other classifiers in both the hybrid and the baseline approaches. Notable improvements include increased specificity (0.824 → 0.878), precision (0.892 → 0.925), and Cohen’s Kappa (0.662 → 0.754), indicating increased reliability and a reduction in false positives. Even weaker-performing models in the baseline setting (e.g. GLM, GBM) benefit significantly from the enriched input.

Although the proposed approach yields promising results, the study has certain limitations that will be addressed in future work. These limitations include the use of median imputation for handling missing data. Although the proportion of missing values was relatively low (<10%) in our dataset, this strategy, despite being simple and computationally efficient, may not fully preserve the intrinsic variability and complexity of the clinical data. Consequently, some patient-specific information and subtle data patterns may have been partially overlooked. Future studies will investigate more advanced imputation approaches to better retain the underlying data structure and potentially improve model robustness and predictive performance. In addition, the study was designed at the hospital-admission level, meaning that admissions from the same patient could potentially appear in different train-test partitions. This may introduce some residual dependence between training and test samples and could slightly overestimate model performance. Future studies will address this issue using patient-level splitting strategies.

Another limitation is the use of MIMIC-IV, which includes only patients in the ICU. This may restrict the generalizability of our findings to broader clinical populations or different care settings. To improve external validity, we intend to validate the proposed approach on multi-center datasets that encompass more heterogeneous patient populations and diverse clinical settings. Additionally, since feature selection in this study was guided solely by clinical expertise, future work will integrate this process with automated selection algorithms such as LASSO, Recursive Feature Elimination, and SHAP to further enhance the model’s robustness and generalizability.

As future work, we plan to extend the framework to: (i) enable patient risk stratification (e.g. low, medium, high risk), thereby supporting more informed clinical decision-making and personalized care pathways; and (ii) investigate the integration of domain-specific large language models (LLMs), including biomedical and clinical models such as ClinicalBERT and BioGPT, to further enhance the predictive capability and clinical applicability of the proposed framework.

## Data Availability

The data used in this study were obtained from the publicly available MIMIC-IV database (version 3.1), accessible through PhysioNet at https://physionet.org/content/mimiciv/3.1/. Access to the database requires completion of the required credentialing process and data use agreement.
